# A comparative approach to decipher intestinal animal-microbe associations

**DOI:** 10.15698/mic2018.11.658

**Published:** 2018-10-30

**Authors:** Keisuke Nakashima

**Affiliations:** 1 Marine Genomics Unit, Okinawa Institute of Science and Technology Graduate University, 1919-1 Tancha, Onna-son, Okinawa 904-0495, Japan.

**Keywords:** gut, microbiota, mucosal immunity, co-evolution, chitin, mucus layer, chordates

## Abstract

Mammalian guts harbor indigenous microbes that are integral to host health. Microbiome research using sophisticated model organisms has provided insights into intricate interactions between microbiota and host animals. However, it remains unclear how these animal-microbe associations developed. We have recently addressed this question via comparative analyses of chordates, given that complex biological systems can be resolved into ancestral and derived elements when examined in an evolutionary framework (Nat Commun 9: 3402). Results support the view that microbial colonization of the mucus layer that overlies mammalian gastrointestinal epithelium was established upon loss of ancestral chitin-based barrier immunity. Comparative approaches enable us to arrange ongoing biological processes into host natural history for better understanding of intestinal animal-microbe associations.

Mammalian intestines are home to indigenous microbial communities. High-throughput sequencing approaches revealed that these microbes are taxonomically diverse and distinct from those of the surrounding environment. Omics analyses of germ-free animals, combined with manipulation of gut flora, provided growing evidence for complex cellular and molecular mechanisms that underlie reciprocal interactions between microbiota and hosts. Nonetheless, we know little about how these intimate animal-microbe associations developed. Biological systems result from a series of evolutionary processes. Thus, in principle, regardless of their complexity, they can be discriminated into ancestral and derived elements when examined in a proper biological setting. Accordingly, we have conducted comparative analyses of chordates.

In animal phylogeny, chordates form a branch of deuterostomes and consist of two invertebrate groups, lancelets and tunicates, as well as vertebrates. Gill slits are a key anatomical feature of deuterostomes, which enable water flow from the mouth to the gill slits. This water flow through the pharynx was prerequisite for their particulate feeding. Invertebrate chordates further employ unique net-like structures secreted from the endostyle, a chordate-specific pharyngeal glandular organ, to establish efficient filter-feeding. Using tunicates, we addressed if this chordate lineage that feeds on environmental microbes harbors indigenous microbes in their gut space similarly to mammals.

Transmission electron microscopy of gut sections of the tunicate *Ciona intestinalis* Type A revealed that the gut space was radially compartmentalized by an unknown membranous structure. Microbes captured by filtration were confined to a luminal space enclosed by this membrane, while a peripheral space over the gut epithelium appeared almost free of bacteria. A combination of chemical and physical analyses demonstrated that the membrane was formed of multilayered, meshed chitin nanofibers embedded in a proteinaceous matrix. The chitinous membranes were secreted and delaminated from the gut epithelium. When chitin synthesis was chemically inhibited, the membrane and axenic space disappeared and food microbes directly contacted with the enterocytes, thereby causing an increased death rate of *Ciona*. These data showed that the endogenous chitinous membranes are essential for formation of the axenic space and contribute to gut homeostasis by acting as a physical barrier (Fig. 1A).

**Figure 1:**
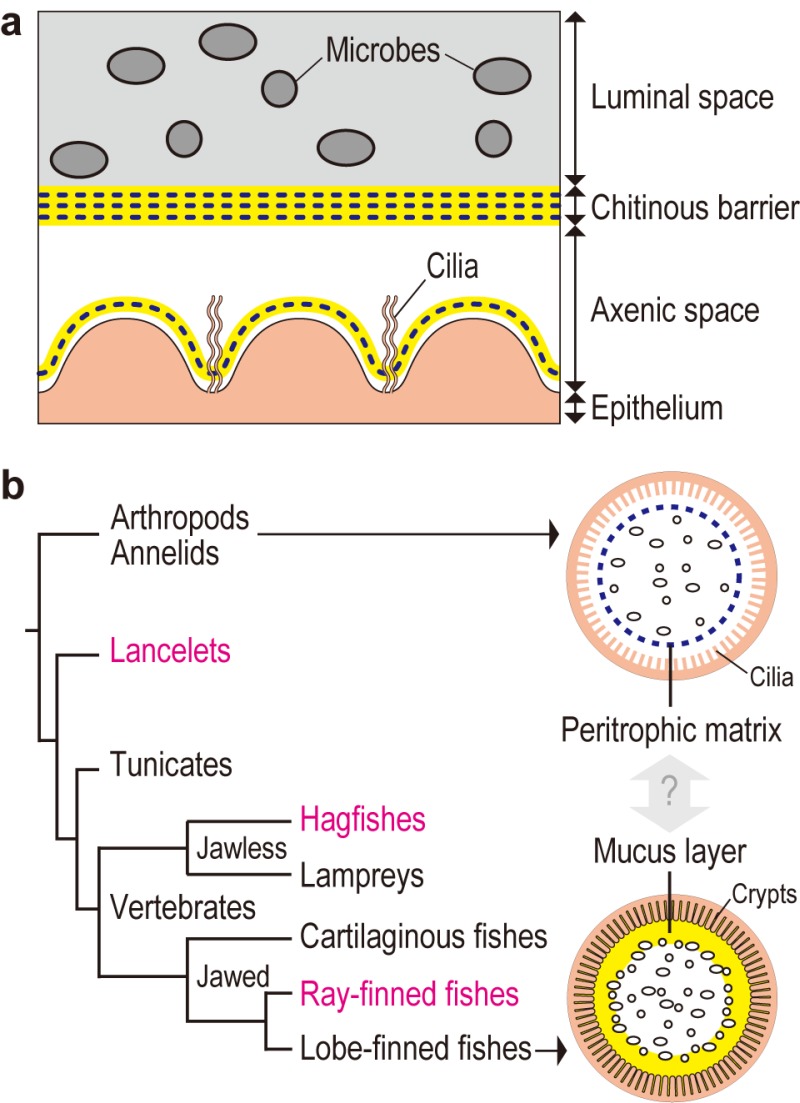
FIGURE 1: Chitin-based barrier immunity. **(A)** A model for the tunicate *Ciona intestinalis* Type A. The gut space is radially compartmentalized into luminal space, to which filter-fed microbes are confined, and peripheral axenic space over the ciliated epithelium, defined by multi-layered chitinous membranes. Chitinous membranes are framed with meshed chitin nanofibers (blue dotted line) embedded in proteinaceous matrix containing gel-forming mucin (yellow). Delamination of a new membrane from the epithelium renews axenic conditions. **(B)** Evolutionary implications. The tree diagram at left depicts phylogenetic relationships of chordates. Two arrows extending from invertebrate outgroups and lobe-finned fishes, which include mammals, point to schematic drawings of intestinal barrier immunity representative of each group. The *Ciona* chitinous barrier **(A)** provides molecular evidence for a possible link between the invertebrate peritrophic matrix (blue dotted line) and the mammalian mucus layer (yellow). To test this idea, animal groups that occupy intervening phylogenetic positions (typed in magenta) are critical. Adapted from Nakashima *et al.* Nat Commun 9: 3402.

Similar chitinous barrier membranes have been well studied in several invertebrate lineages. So-called invertebrate peritrophic matrices structurally resemble the *Ciona* membrane, as these structures are framed with meshed chitin nanofibers produced by homologous chitin synthases. On the other hand, these structures have distinct matrix protein compositions. While invertebrate peritrophic matrix consists of protein with chitin-binding and/or proline-threonine-serine-domains, *Ciona* membranes are comprised of anti-bacterial proteins and gel-forming mucin, which is the main constituent of the mammalian mucus layer that covers the gut epithelium. Briefly, the *Ciona* membrane provides molecular evidence for a possible link between the invertebrate peritrophic matrix and the mammalian mucus layer, which are considered analogous, i.e. with no common descent (Fig. 1B). To test this idea, we have examined the following chordate lineages that occupy phylogenetic positions between invertebrates and mammals: a basal chordate *Branchiostoma floridae* (lancelet), a jawless vertebrate *Eptatretus atami* (hagfish), and a jawed vertebrate *Oreochromis mossambicus* (ray-finned fish). All these organisms possessed intestinal chitinous membranes.

The finding of chitinous membranes in the ray-finned fish, *O. mossambicus*, seemingly contradicts the common view that gut mucosal surfaces of ray-finned fish are covered with a mucus layer that is colonized by indigenous microbes as in mammals. Actually, 16S ribosomal RNA gene analysis showed the presence of indigenous microbes in the gut of *O. mossambicus*, but this microbial community was enclosed by chitinous membranes and was segregated from the surrounding mucus layer secreted by crypt goblet cells. In contrast, we were unable to detect chitin in the guts of mice, where gut microbiota directly interact with goblet cell-derived mucus.

In conclusion, this comparative study provides a glimpse of evolutionary changes in the intestinal mucosal surfaces of chordates. We proposed a transition from a chitin-based ancestral condition to a mucin-based derived state (Fig. 2). Radial compartmentalization of the gut space by chitinous membrane seems to be an ancestral feature of chordates. In tunicates, filter-fed microbes transiently pass through the intestine, while being confined to the luminal space by the chitinous barrier. Ray-finned fishes retain this chitin-based barrier immunity, but viable passage of ingested microbes was prevented by bactericidal gastric juice, an invention of jawed vertebrates. This would have offered the subsequent gut space as a new ecological niche for surviving microbes. Because mammals lack the chitinous barrier, gut microbes interact directly with the surrounding goblet cell-derived mucus. Thus, it seems that chitin-based barrier immunity and its loss predated mucus colonization by indigenous gut microbiota.

**Figure 2:**
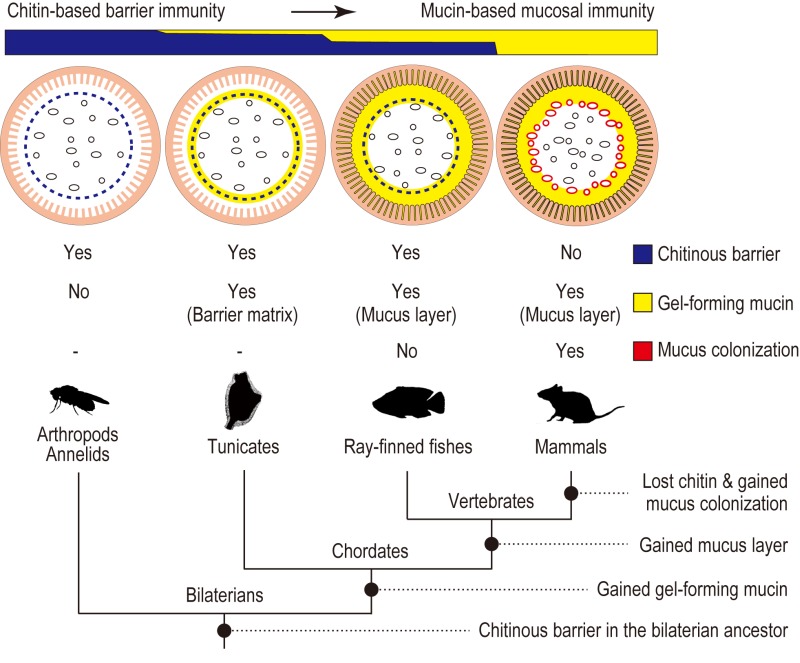
FIGURE 2: Transition of the gut mucosal surface in chordates. For animal groups shown in pictograms, intestinal barrier structures are illustrated above. Gut microbes are shown as ovals and circles. Invertebrate outgroups share a peritrophic matrix, although the presence of chitin remains unclear beyond arthropods and annelids. Tunicates possess chitin nanofibers embedded in matrix substances, including gel-forming mucins. Filter-fed microbes are confined to the luminal space. Ray-finned fishes harbor indigenous gut microbes that are separated from the surrounding mucus layer by chitinous barriers. Mammals lost chitin, and the mucus layer is colonized by indigenous microbes. “Yes” or “No” indicates the presence or absence of the item listed on the right, respectively. Color-codes are shown in boxes. The bottom tree diagram depicts animal phylogeny, annotated with a series of events that account for gradual changes of the gut mucosa in chordates. We propose a transition from a chitin-based ancestral condition to a mucin-based derived state (top). Adapted from Nakashima *et al.* Nat Commun 9: 3402.

There are considerable variations in intestinal mucus-microbe interactions. In mice, ileum mucus is loose, yet it limits microbial access to host tissues through joint actions with diffusible molecules like RegIIIγ. Colon mucus forms two layers with the outer layer extensively colonized by microbes and the densely packed inner layer devoid of microbes. The degree of mucus penetrability varies among bacterial species. These variations can be explained in the context of regional adaptation driven by local functional demands since the onset of direct mucus-microbe interaction. Accordingly, comparative approaches enable us to arrange ongoing biological processes into host natural history to decipher the complex animal-microbe association.

